# Expression of SH3 and Multiple Ankyrin Repeat Domains Protein 3 in Mouse Retina

**DOI:** 10.3389/fncel.2022.795668

**Published:** 2022-03-25

**Authors:** Yue Xu, Ya’nan Wang, Guang’an Tong, Lin Li, Juan Cheng, Lesha Zhang, Qi Xu, Liecheng Wang, Pingping Zhang

**Affiliations:** ^1^Department of Physiology, School of Basic Medical Sciences, Anhui Medical University, Hefei, China; ^2^Department of Neurology, The Affiliated Hospital of the Neurology Institute, Anhui University of Chinese Medicine, Hefei, China

**Keywords:** ASD, SHANK3, retina, double-labeled immunohistochemistry, excitatory synapse

## Abstract

Synapse-associated gene mutations of SH3 and multiple ankyrin repeat domains protein 3 (SHANK3) may lead to autism spectrum disorder (ASD). In some ASD cases, patients may also have vision disorders. However, the effects of SHANK3 in the retina are barely mentioned in the literature. In this study, we used wild-type mice to systematically map the distribution of SHANK3 expression in entire mouse retinas. Using Western blot analysis and immunofluorescence double labeling, we identified a large number of prominent cells expressing high levels of SHANK3 in the inner retina, in particular, the ganglion cell layer (GCL) and inner nucleus layer. The inner plexiform layer and outer nucleus layer were also exhibited positive SHANK3 signals. In the inner layer, GABAergic amacrine cells (ACs) labeled by glutamate decarboxylase were colocalized with SHANK3-positive cells. Dopaminergic ACs (labeled by tyrosine hydroxylase) and cholinergic ACs (labeled by choline acetyltransferase) were also found to contain SHANK3-positive signals. Additionally, most GCs (labeled by Brn3a) were also found to be SHANK3 positive. In the outer retina, bipolar cells (labeled by homeobox protein ChX10) and horizontal cells (labeled by calbindin) were SHANK3 positive. In the outer nucleus layers, the somata of blue cones (labeled by S-opsin) were weekly co-labeled with SHANK3. The inner segments of blue cones and the outer segments of red/green cones (labeled by L/M-opsin) were partially colocalized with SHANK3 and the outer segments of rods (labeled by Rho4D2) were partially SHANK3 positive too. Moreover, SHANK3-positive labeling was also observed in Müller cells (labeled by cellular retinaldehyde-binding protein). These wide expression patterns indicate that SHANK3 may play an important role in the visual signaling pathway.

## Introduction

Autism spectrum disorder (ASD) is a neurodevelopmental disease with the core symptoms of social deficit and repetitive behavior (Huerta et al., 2012; Lutz et al., 2020). Numerous studies have indicated that synaptic-associated gene mutations may cause ASD, including fragile X mental retardation 1 (FMR1) (Fragile X syndrome), tuberous sclerosis gene (TSC) (tuberous sclerosis), SH3, and multiple ankyrin repeat domains 3 (SHANK3) [Phelan–McDermid syndrome (PMS)] (Zoghbi and Bear, 2012). A known cause of PMS is the dysfunction of SHANK3 (Phelan, 2008; Costales and Kolevzon, 2015; Tachibana et al., 2017; Mitz et al., 2018) and patients with PMS commonly display typical autism features, with some studies showing that these patients may also have skeletal muscle hypotonia, intellectual disability, developmental delay, and visual problems (Phelan et al., 2001, 2005; Phelan, 2008; Phelan and McDermid, 2012). SHANK genes encode three distinct proteins (SHANK1-3) (Sheng and Kim, 2000) and contain five domains/regions that are involved in protein–protein interactions. As an excitatory postsynaptic scaffold protein, SHANK3 plays an important role in anchoring scaffolding complexes to the cytoskeleton of postsynaptic densities (Bozdagi et al., 2010; Verpelli et al., 2011; Halbedl et al., 2016) and interacts with various postsynaptic density proteins.

Only a few studies have focused on the cellular localization of SHANK1-3 in the retina. Specifically, SHANK1 expression has been found in the retina of the Xenopus (Gessert et al., 2011). In adult mouse retinas, SHANK1A was found to be widely expressed in the inner plexiform layer (IPL) and outer plexiform layer (OPL), restrictedly located in the cone pedicles—not the rod spherules (Stella et al., 2012). The expression difference of SHANK1A between the cone and rod photoreceptors may indicate the different effects of SHANK1A in the cone and rod signaling pathways. Brandstätter has reported that SHANK2 were expressed in retinal synapses (Brandstätter et al., 2004), with the further study indicating that, in developing retinas, SHANK2 can be found in the IPL as well as the ganglion cell layer (GCL), which is related to neuronal differentiation (Kim et al., 2009). This means that SHANK2 may play a critical role in the neuronal differentiation of the developing retina. It is well-known that patients with PMS have visual problems and Tatavarty’s work (Tatavarty et al., 2020) also shows that SHANK3 is essential for homeostatic plasticity in the mouse visual cortex, suggesting that there is a critical effect of SHANK3 in the visual signaling pathways (Phelan et al., 2005; Phelan, 2008; Phelan and McDermid, 2012). Although SHANK3 plays an important role in the visual system, its cellular localization patterns in mice retina are still not well understood.

In this study, using double-labeled immunohistochemistry, we systematically analyzed the expression pattern of SHANK3 in mice retina. Our results suggest that SHANK3-positive signals are widely found in mouse retinas, including the cones, rods, horizontal cells (HCs), several subtypes of amacrine cells (ACs), bipolar cells (BCs), ganglion cells (GCs), inner/outer plexiform layers, and Müller cells. Therefore, these extensive expressions of SHANK3 indicate that it may participate in lateral and longitudinal retinal information regulation.

## Materials and Methods

### Ethics Statement

All the animal experiments were performed in accordance with the guidelines of the China Council on Animal Care and Use. This study was approved by the National Institutes of Health (NIH) guidelines for the Care and Use of Laboratory Animals and the regulations of Anhui Medical University regarding the ethical use of animals. All the efforts were made to minimize the number of animals used and their suffering. The animal experiments were performed at Anhui Medical University (Hefei, China). Mouse eyes were used in this study, with all the mice deeply anesthetized with 20% urethane (10 ml/kg) before the eyes were enucleated; the mice were then sacrificed *via* CO2 asphyxiation.

### Animals

Wild-type C57BL6/J (WT) mice were purchased from the Anhui Medical University Animal Center. Shank3b KO mice were purchased from the Jackson Laboratory (Catalog #017688). All the animal procedures were approved by the Animal Experiment Committee of Anhui Medical University. Three to five mice (6–8 weeks old) were housed per cage at a temperature of 23 ± 2°C under a 12-h light and dark cycle and were provided food and water *ad libitum*.

### Deoxyribonucleic Acid Analysis and Genotyping

The genotypes of WT mice and *Shank3b^–/–^* mice were determined by PCR of mouse tail DNA, using two primers: primer F1b (GAGCTCTACTCCCTTAGGACTT) and R1b (TCCCCCTTTCACTGGACACCC) for the wild-type allele (316 base pairs) and F1b and R2 (TCAGGGTTATTGTCTCATGAGC) for the mutant allele (360 base pairs). The genotype data were shown in Supplementary Figure 1.

### Ribonucleic Acid Isolation and Reverse Transcription

Total RNA was extracted from wild-type mouse and shank3b ko mouse using SPARKeasy Tissue and Cell RNA Rapid Extraction Kit (Sparkjade, Shandong, China, AC0202) according to the manufacturer’s instructions. The RNA concentration was determined using a NanoDrop 1,000 Micro Volume Spectrophotometer (Thermo Fisher Scientific, United States). Every 1 μg of the total RNA was reverse transcribed into cDNA in a 20-μl system of the SPARKscriptA II RT Plus Kit (With gDNA Eraser) (Sparkjade, AG0304) according to the manufacturer’s instructions.

### Reverse Transcription-Quantitative PCR

The expression levels of SHANK3 mRNA were evaluated by reverse transcription-quantitative PCR (RT-qPCR) using GAPDH as the internal control. The generated cDNAs were used for qPCR using PerfectStart Green qPCR SuperMix (TransGen Biotech, Beijing, China, AQ601) according to the manufacturer’s protocol. The qPCR process was performed using a two-step method in a 20 μl total reaction volume using a CFX 96 Real-Time system (Bio-Rad, California, United States). The following thermocycling conditions were used: 94°C for 30 s, followed by 45 cycles of 94°C for 5 s, 60°C for 15 s, and 72°C for 10 s followed by a final extension at 72°C for 40 s. The primer sequences were as follows: SHANK3 forward, 5′- ACCTTGAGTCTGTAGATGTGGAAG -3′ and reverse, 5′- GCTTGTGTCCAACCTTCACGAC -3′ and GAPDH forward, 5′- CATCACTGCCACCCAGAAGACTG -3′ and reverse, 5′- ATGCCAGTGAGCTTCCCGTTCAG -3′. The relative mRNA expression of the target gene in each sample was calculated using the quantification cycle (Cq) value based on the 2^–ΔΔCq^ method. The RT-qPCR data were shown in Supplementary Figure 1 and Supplementary Table 1.

### Tissue Preparation for Immunocytochemistry

The retinas were prepared as previously described in detail (Zhang et al., 2020) with minor modifications. The mice were deeply anesthetized *via* 20% Urethane and transcardially perfused with saline followed by 4% paraformaldehyde (both ice-cold). After perfusion, the eyes enucleated from the animals were immediately placed in 4% buffered paraformaldehyde for 15 min. The eyecups were carefully removed from the eyes and then chilled sequentially in 10 (w/v), 20, and 30% sucrose in 0.1 M phosphate-buffered saline (PBS) (pH 7.4) at 4°C. The eyecups were then embedded in optimum cutting temperature (OCT) compound (Sakura Finetek, Torrance, Japan), frozen in liquid nitrogen, and sectioned vertically with a 14-μm thickness on a freezing microtome (Leica, Nussloch, Germany). The sections were then mounted on gelatin chromium-coated slides.

### Immunocytochemistry

The immunocytochemistry procedures were performed according to a method described previously (Sheng et al., 2021). Briefly, the retinal sections were preincubated in 0.1 M PBS containing 6% normal donkey serum, 1% bovine serum albumin, and 0.2% Triton X-100 (PBST) for 2 h at 4°C. Rabbit polyclonal antibody against mouse SHANK3 (corresponding to amino acid residues 841–855) (1:1,000 dilution, APZ-013, Alomone Labs, Israel) was used for labeling the SHANK3. And a blocking peptide (BLP-PZ013, Alomone labs) for anti-Shank3 antibody was used as a negative control. The experiments were conducted by double-labeling. All the antibodies were mixed with PBST. The sections were combined with primary and secondary antibodies sequentially. The primary antibodies used in this work were as follows: goat anti-S-opsin antibody (1:500 dilution, sc-14363, Santa Cruz Biotechnology, United States), goat anti-L/M-opsin antibody (1:800 dilution, sc-22117, Santa Cruz Biotechnology, United States), and mouse anti-Rho4D2 antibody (1:1,500 dilution, ab98887, Abcam plc., United Kingdom), were used for labeling the blue cones, red/green cones, and rods, respectively. Mouse anticalbindin D-28k (CB) monoclonal antibody (1:2,000 dilution, Swant, Switzerland) and sheep antihomeobox protein ChX10 (ChX10) polyclonal antibody (1:800 dilution, Abcam, United Kingdom) were used for labeling the HCs and BCs, respectively. The antibodies used for labeling the different AC subtypes were as follows: mouse antiglutamate decarboxylase 65 (GAD 65) monoclonal antibody (1:1,000 dilution, Abcam) for GABAergic ACs, mouse anti-tyrosine hydroxylase (TH) monoclonal antibody (1:10,000 dilution, Sigma Aldrich, United States) for dopaminergic ACs, and sheep anticholine acetyltransferase (ChAT) polyclonal antibody (1:1,000 dilution, Millipore, United States) for cholinergic ACs. Mouse anti-Brn3a monoclonal antibody (1:500 dilution, Santa Cruz, United States) was used for labeling the GCs and mouse anticellular retinaldehyde-binding protein (CRALBP) monoclonal antibody (1:1,000 dilution, Abcam) was used for labeling the Müller cells. Additionally, Mouse anti-PSD95 monoclonal antibody (1:500 dilution, GTX634291, GeneTex, United States) was used for labeling postsynaptic density protein 95 (PSD95), Mouse antisynaptophysin monoclonal antibody (1:500 dilution, 67864-1-Ig, Proteintech, China) was used for labeling presynaptic membrane, and Guinea pig anti-vesicular glutamate transporter 1 (VGluT1) polyclonal antibody (1:2,000 dilution, Chemicon, United States) was used for labeling glutamatergic synapses.

The secondary antibodies used in this study were as follows: Alexa Fluor 488-conjugated donkey anti-rabbit IgG (1:50 dilution, Jackson, United States) for labeling the SHANK3; Alexa Fluor 555-conjugated donkey anti-mouse IgG (1:100 dilution, Abcam) for CB, GAD 65, TH, Brn3a, CRALBP, rods, PSD95 and Synaptophysin; Alexa Fluor 555-conjugated donkey antigoat IgG (1:100 dilution, Abcam) for S-opsin and L/M-opsin; Alexa Fluor 555-conjugated donkey antisheep IgG (1:100 dilution, Abcam) for ChX10 and ChAT and Alexa Fluor 594 conjugated donkey antiguinea pig IgG (1:100 dilution, Jackson, United States) for VGluT1. Control experiments included the omission of primary and/or secondary antibodies or preabsorption with antigenic blocking peptide for SHANK3 (BLP-PZ013, Alomone Laboratories).

### Confocal Laser Scanning Microscopy

The sections were scanned with a ZEISS LSM880 confocal laser scanning microscope (ZEISS, LSM880 + Airyscan, Germany) using a 40X oil immersion objective lens. For each double-labeling experiment, a total of 30–36 sections on six different glass slides derived from three or four eyeballs were examined. Single optical sections were taken *via* preparation and recorded digitally. To avoid any possible reconstruction stacking artifact, the double labeling was precisely evaluated using sequential scanning of the single-layer optical sections. The images were resized and adjusted for brightness and contrast in Adobe Photoshop to reproduce the original histological data.

### Western Blot Analysis

Western blot analysis was performed as described previously in detail (Sheng et al., 2021). Mice retinal extract samples were loaded, subjected to 8% sodium dodecyl sulfate/polyacrylamide gel electrophoresis (SDS/PAGE), and then transferred onto polyvinylidene fluoride (PVDF) membranes (Merck Millipore, Ireland). Non-specific binding was blocked for 2 h at room temperature in a blocking buffer consisting of 20 mM Tris/HCl, pH 7.4, 137 mM NaCl, 0.1% Tween-20, and 5% non-fat milk. The blots were incubated with the anti-SHANK3 antibody (1:1,000 dilution, Alomone labs) overnight at 4°C, followed by horseradish peroxidase-conjugated goat antirabbit IgG (1:20,000 dilution, ZSGB-BIO, Beijing, China) for 2 h at room temperature, and finally visualized with an enhanced chemiluminescence automatic gel imaging analysis system (Peiqing Science and Technology, Shanghai, China). No band was detected when the SHANK3 antibody was preabsorbed with the immunizing peptide for 3 h at room temperature. To estimate the molecular weight (MW) of SHANK3, a prestained marker (Tiangen, Beijing, China) was used.

## Results

### Expression Profile of SH3 and Multiple Ankyrin Repeat Domains Protein 3 in Mouse Retinas

**FIGURE 1:**
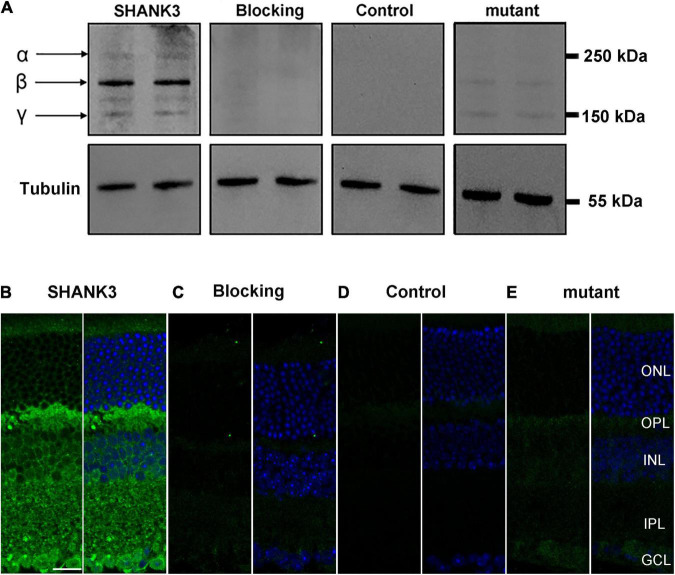
Expression of SH3 and multiple ankyrin repeat domains protein 3 (SHANK3) in mice retina. **(A)** Western blot analysis confirmed that SHANK3 was specifically expressed in the retinal homogenates, with three SHANK3 isoforms at 150–250 kDa. No band was detected when the SHANK3 antibody was pre-absorbed with the immunizing antigen. The similar was find when the primary antibody was omitted. And when the shank3 was mutant, the bands of SHANK3 were reduced. **(B)** Confocal fluorescence microphotographs of a vertical section of the mouse retina, labeled by SHANK3. Fluorescence staining shows that the SHANK3 is widely distributed through the whole retina. **(C)** Micrographs of a retinal vertical section, showing that no signal was detectable when the primary antibody for SHANK3 was pre-absorbed with the immunizing antigen. **(D)** Micrographs of a retinal vertical section, showing that no signal was detectable when the primary antibody was omitted. **(E)** Micrographs of a retinal vertical section, showing that the shank3 was mutant, the signal of SHANK3 was reduced. ONL, outer nuclear layer; OPL, outer plexiform layer; INL, inner nuclear layer; IPL, inner plexiform layer; GCL, ganglion cell layer. Scale bar = 20 μm.

The specificity of SHANK3 was examined using Western blot analysis. As shown in Figure 1A in the mice retinal homogenates, the antibody against the SHANK3, recognized three bands at 150–250 kDa—termed α, β, and γ—consistent with previous reports (Mei et al., 2016). A negative reaction when the primary antibodies were blocked with a specific blocking peptide (BLP-PZ013, alomone labs) for anti-Shank3 antibody. Additionally, immunostaining for SHANK3 was eliminated when the primary antibodies were omitted. Meanwhile, when the *shank3* was mutant, the immunoreaction (IR) of SHANK3 was reduced, this result was the same as the earlier receptor (Peça et al., 2011). Quantitative PCR analysis of SHANK3 mRNA in WT and shank3b knockout mice revealed that SHANK3 mRNA indeed existed in the WT mice (Supplementary Figure 2 and Supplementary Table 1). These results suggest that SHANK3 was indeed present in mouse retinas.

To determine the spatial expression pattern of SHANK3 within the retina, antibody against mouse SHANK3 was used. In vertical cryostat sections of the mouse retinas (Figure 1B), the IR of SHANK3 was mainly observed in the inner retina, including the OPL and IPL, with the labeling diffusely distributed in the OPL and throughout the full thickness of the IPL. Moreover, the IR of SHANK3 was clearly observed in many cells in the INL. Based on the positions and shapes of these cells, they could be HCs, BCs, or ACs. Numerous cells in the GCL could be displaced ACs or GCs. When the antibody was preabsorbed (negative control), only a low level of the background was detected (Figure 1C), which was similar to the no primary antibody control (Figure 1D) and *Shank3*-mutant control (Figure 1E), suggesting that the labeling was specific.

### SH3 and Multiple Ankyrin Repeat Domains Protein 3 Is Expressed in Photoreceptors

**FIGURE 2:**
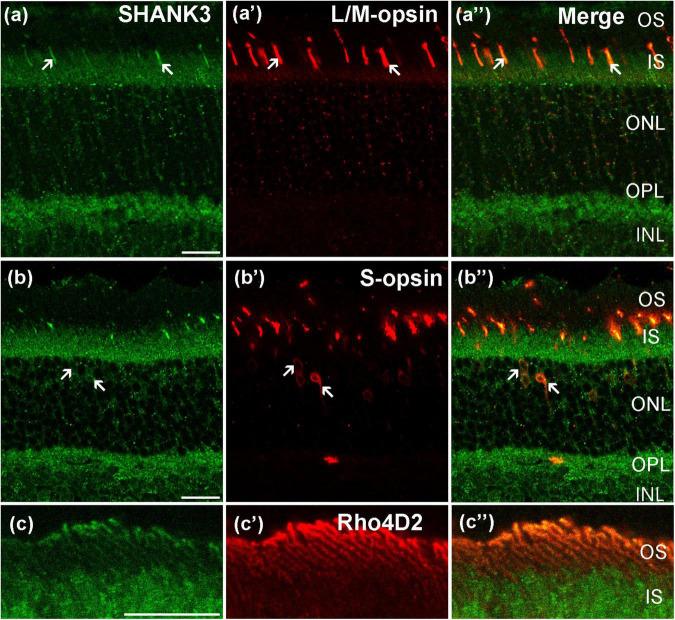
Confocal microphotographs showing the distribution of SHANK3 in the photoreceptors. Double-labeled elements (green for SHANK3; red for L/M-opsin, S-opsin and Rho4D2) appear yellowish and some double-labeled cells are indicated by arrows. **(a–a”)** Colocalization of SHANK3 with L/M- opsin in a retinal vertical section. Fluorescence double stainings show that SHANK3 **(a)** and the L/M-opsin **(a’)**, the merged image of L/M-opsin and SHANK3 **(a”)**. L/M-opsin-labeled the outer segment (OS) of red/green cones **(a’)**. Part of the L/M-opsin-positive IS (arrows) are co-labeled by SHANK3 **(a,a”)**. **(b–b”)** Colocalization of SHANK3 with S-opsin in a retinal vertical section. Fluorescence double stainings show that SHANK3 **(b)** and the S-opsin **(b’)**, and **b”** is the merged image of S-opsin and SHANK3. S-opsin is a marker for blue cone. Note that labeling for SHANK3 is detected in S-opsin-positive blue cones (arrows). **(c–c”)** Colocalization of SHANK3 with Rho4D2 in a retinal vertical section. Fluorescence double stainings show that SHANK3 **(c)** and the Rho4D2 **(c’)**, the merged image of Rho4D2 and SHANK3 **(c”)**. Rho4D2-labeled the outer segment (OS) of rods **(c’)**. Rho4D2-positive OS are partially labeled by SHANK3 **(c,c”)**. OS, outer segment; IS, inner segment. Scale bar = 20 μm.

To investigate the expression of SHANK3 in the photoreceptors layer, we performed double-labeling experiments. Figures 2a–a” shows localization of the SHANK3 in red/green cones. Double labeling revealed that the SHANK3 immunoreactivity was only found on the L/M-opsin-labeled outer segment (OS) of the red/green cone (81 out of 195 positive segments from four sections, 40.9% positive rate, Sheng et al., 2021). Figures 2b–b” shows the localization of the SHANK3 in blue cones. Double labeling shows the SHANK3 signals were weekly expressed on the inner segment (IS) and somata of S-opsin-labeled blue cones (481 somat out of 481 cells from four sections, 100% positive rate, Sheng et al., 2021). Figures 2c–c” shows the localization of the SHANK3 in rods. Double labeling shows the SHANK3 signals were weekly expressed on the OS of Rho4D2-labeled blue cones (521 out of 521 positive segments from four sections, 100% positive rate, Sheng et al., 2021).

### SH3 and Multiple Ankyrin Repeat Domains Protein 3 Is Widely Expressed in the Outer Retina

**FIGURE 3:**
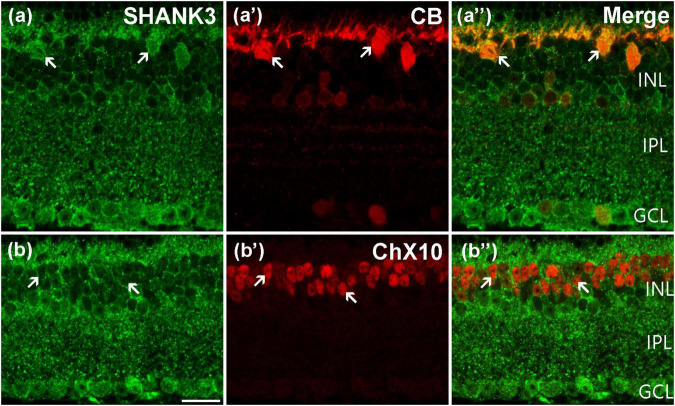
Confocal microphotographs showing the distribution of SHANK3 in the outer retina. Double-labeled elements (green for SHANK3; red for CB and ChX10) appear yellowish and some double-labeled cells are indicated by arrows. **(a–a”)** Colocalization of SHANK3 with CB in a retinal vertical section. Fluorescence double stainings show that SHANK3 **(a)** and the CB **(a’)**, the merged image of CB and SHANK3 **(a”)**. CB-labeled horizontal cells **(a’)**. The CB-positive somata (arrows) are colabeled by SHANK3 **(a,a”)**. **(b–b”)** Colocalization of SHANK3 with ChX10 in a retinal vertical section. Fluorescence double stainings show that SHANK3 **(b)** and the ChX10 **(b’b”)** is the merged image of ChX10 and SHANK3. ChX10 is a marker for bipolar cells. Note that labeling for SHANK3 is detected in ChX10-positive BCs (arrows). Scale bar = 20 μm.

Using immunohistochemistry double-labeling experiments, we explored the expression pattern of SHANK3 in the inner mouse retinas. The general labeling profile of SHANK3 in a vertical section of a mouse retina (green) is shown in Figure 3a and a specific marker (CB) for rodent HCs, used for labeling the HCs (red), is shown in Figure 3a’ (Chandra et al., 2019; Zhang et al., 2019), the merged figures of green and red signals is shown in Figure 3a”. As revealed, the somata and processes of the CB-positive HCs (arrows) are located at the distal margin of the INL (Figure 3a’). In the merged image of Figure 3a”, the CB-positive HCs are clearly labeled by SHANK3, especially in the cytoplasm. A total of 64 CB-labeled neurons in 25 retinal slices were identified in the outer INL, and they were colabeled by SHANK3 (64 out of 64 cells, 100% positive rate).

Using antibody against ChX10, which is a specific BCs marker, shows that ChX10-positive BCs are located in the outermost portion of the INL (Figure 3b’; Cui et al., 2020; Sheng et al., 2021). When the sections are co-stained with SHANK3 (Figures 3b,b”), the soma of the ChX10-positive BCs was prominently embedded within the SHANK3-positive BCs’ membranes (arrows in Figure 3b”). Our results demonstrated 1153 ChX10-positive neurons in 17 retinal slices appeared to be SHANK3-positive (1,153 out of 1,153 cells, 100% positive rate). Thus, the BCs are also shown to express SHANK3, especially in the plasma membrane.

### SH3 and Multiple Ankyrin Repeat Domains Protein 3 Is Widely Expressed in the Inner Retina

**FIGURE 4:**
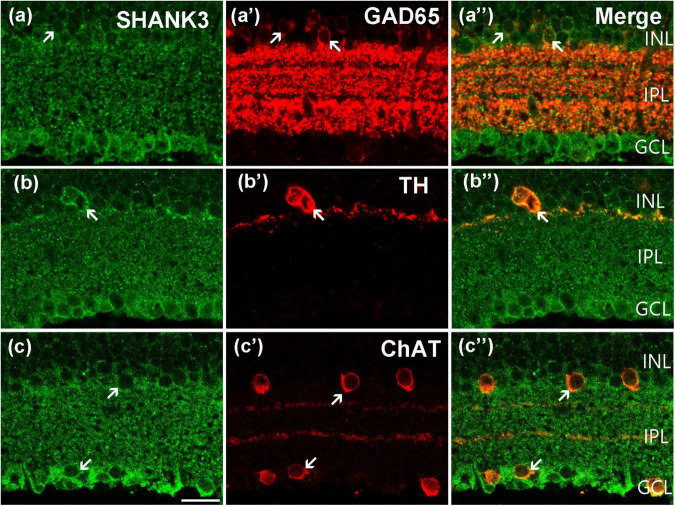
Confocal microphotographs showing the distribution of SHANK3 in different AC subtypes. Double staining (green for SHANK3; red for GAD 65, TH, and ChAT) appear yellowish and arrows indicate some double-labeled cells. **(a–a”)** Colocalization of SHANK3 with GAD 65 in a retinal vertical section. Fluorescence double stainings show that SHANK3 **(a)** and the GAD 65 **(a’)**. Note that the GABAergic ACs in the innermost part of the INL are GAD 65-labeled, and also strongly labeled numerous neuronal processes of these cells in the IPL **(a’)**. All the GAD 65-positive GABAergic ACs somata are labeled by SHANK3 **(a”)**. **(b–b”)** Colocalization of SHANK3 with TH in a retinal vertical section. Fluorescence double stainings show that SHANK3 **(b)** and the TH **(b’)**. The soma and processes of the dopaminergic ACs are strongly labeled by TH **(b’,b”)**, and the soma is clearly labeled by SHANK3 **(b”)**. **(c–c”)** Colocalization of SHANK3 with ChAT in a retinal vertical section. Fluorescence double stainings show that SHANK3 **(c)** and the ChAT **(c’)**. ChAT-labeled mirror-symmetric cholinergic ACs and their processes, forming two bands in the IPL. The merged image shows that the somata of cholinergic ACs in INL and GCL were both SHANK3-positive **(c”)**. Scale bar = 20 μm.

Using double-labeling experiments, we further explored the co-expression pattern of SHANK3 and the different subtypes of ACs. GABAergic ACs, which account for a majority of ACs in the mammalian retina, are all large-field ACs labeled by GAD 65 (Halbedl et al., 2016; Zhang et al., 2020). As shown in Figure 4a’, the somata of the GAD65-positive GABAergic ACs are located in the inner part of the INL and their processes are widely expressed in the IPL. After double-labeled of GAD65 and SHANK3, the merged image shows that almost all GAD65-positive GABAergic ACs were colabeled by SHANK3 (Figure 4a”). Our results demonstrated that the GAD65-immunolabeled all neurons observed in this study were colabeled by SHANK3 (519 out of 519 cells from 22 retinal slices with the positive rate being 100%). Therefore, the double-labeling experiment confirmed that SHANK3 is expressed in GABAergic ACs.

Dopaminergic ACs are a small subpopulation of GABAergic ACs that can be immune labeled by antibody against TH (Balasubramanian et al., 2018; Ning et al., 2021). TH-positive dopaminergic ACs are located in the inner part of the INL, sending processes mostly into the outmost sublayer of the IPL (Figure 4b’). As shown in the merged image of Figure 4b”, the plasma membrane of the TH-positive ACs was strongly colocalized (arrow) with SHANK3, and the processes of dopaminergic ACs may be SHANK3-positive too. Twenty-four cells with obvious TH immunostaining were observed in 23 retinal slices, and they were all SHANK3-positive (24 out of 24 cells, 100% positive rate).

Cholinergic ACs are a small subgroup of GABAergic ACs that can be stained by antibodies against ChAT (He et al., 2019; Ivanova et al., 2020). Typical cholinergic ACs are arranged in two mirror-symmetric populations, with their processes forming two distinct fluorescence bands in the IPL (Figure 4c’). These cells were also immunoreactive to SHANK3 (Figure 4c”), and both the somata in the INL and GCL were overlapped (arrows in Figure 4c”). In a quantitative analysis involving 224 ChAT-positive neurons collected from 27 retinal slices, all of these ChAT-positive ACs were SHANK3 immunoreactive (224 out of 224 cells, 100% positive rate).

### SH3 and Multiple Ankyrin Repeat Domains Protein 3 Is Expressed in Pre/Post-synaptic Membrane

**FIGURE 5:**
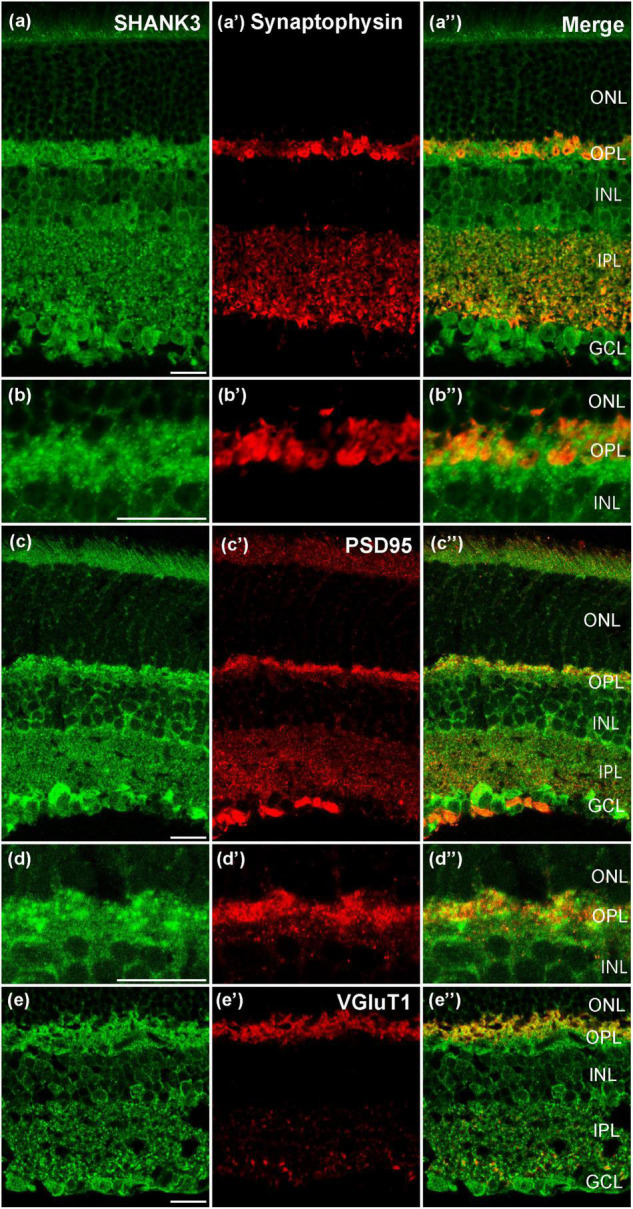
Confocal microphotographs showing the distribution of SHANK3 in synaptic. Double staining (green for SHANK3; red for Synaptophysin, PSD95 and VGluT1) appear yellowish. **(a–a”)** Colocalization of SHANK3 with Synaptophysin in a retinal vertical section. Fluorescence double stainings show that SHANK3 **(a)** and the Synaptophysin **(a’,a”)** is the merged image of Synaptophysin and SHANK3. **(b–b”)** are the locally magnified images for **(a–a”)**. The merged image shows that the presynaptic membrane is not labeled by SHANK3. **(c–c”)** Colocalization of SHANK3 with PSD95 in a retinal vertical section. Fluorescence double stainings show that SHANK3 **(c)** and the PSD95 **(c’,c”)** is the merged image of PSD95 and SHANK3. **(d–d”)** are the locally magnified images for **(c–c”)**. The merged image shows that the postsynaptic membrane is SHANK3-positive. **(e–e”)** Colocalization of SHANK3 with VGluT1 in a retinal vertical section. Fluorescence double stainings show that SHANK3 **(e)** and the VGluT1 **(e’,e”)** is the merged image of VGluT1 and SHANK3. Scale bar = 20 μm.

To explore the expression of the SHANK3 in pre-/postsynapse, we performed fluorescent labeling studies using the SHANK3 antibody together with specific presynaptic marker Synaptophysin, (Chang et al., 2020). The Synaptophysin was widely expressed in the OPL and IPL (Figures 5a’,b’), and the IR of OPL is stronger than IPL. As the magnified figures show that the SHANK3-positive OPL neurons’ nobs were not colabeled with Synaptophysin (Figures 5a–a”, magnified Figures 5b–b”). Then, we performed the double-labeling study between PSD95 (a specific marker for postsynaptic membrane) and SHANK3 (Takada et al., 2008). The IR of SHANK3 was found in PSD-95-positive OPL and IPL (Figures 5c”,d”). Figures 5d–d” was the magnified parts from Figures 5c–c”. We also performed the double-labeling study between VGluT1 (a specific marker for Glutamatergic synapses) and SHANK3 (Michalski et al., 2013). VGluT1 was expressed on neuronal processes reaching into all sublayers of the IPL and to an even higher level on synaptic terminals in the OPL (Figures 5e–e”), show the significant colocalization with SHANK3. These results suggest that SHANK3 was especially expressed in the postsynaptic membrane.

### SH3 and Multiple Ankyrin Repeat Domains Protein 3 Is Expressed in the Ganglion Cell Layer

**FIGURE 6:**
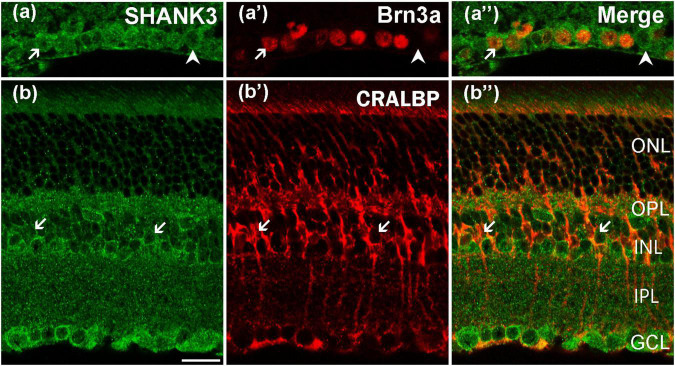
Confocal microphotographs showing the distribution of SHANK3 in GCL and Müller cells. Double staining (green for SHANK3; red for Brn3a and CRALBP) appear yellowish, and arrows indicate some of the double-labeled cells. **(a–a”)** Colocalization of SHANK3 with Brn3a in a retinal vertical section. Fluorescence double stainings show that SHANK3 **(a)** and the Brn3a **(a’)**, **(a”)** are the merged image of Brn3a and SHANK3. Almost all the ganglion cells are clearly labeled by Brn3a **(a’)**. SHANK3 immunoreactivity is observed in all Brn3a (arrows). Meanwhile, some SHANK3-positive cells in the GCL are not immunoreactive for Brn3a (arrowheads in **a–a”**), which could be displaced ACs or Brn3a-negative GCs. **(b–b”)** Colocalization of SHANK3 with CRALBP in a retinal vertical section. Fluorescence double stainings show that SHANK3 **(b)** and the CRALBP **(b’,b”)** is the merged image of CRALBP and SHANK3. CRALBP is a specific Müller cell marker. Note that SHANK3 immunostaining is observed in somata of CRALBP-positive Müller cells. Scale bar = 20 μm.

As previously mentioned, the cells in the GCL could be GCs or displaced ACs. To determine if GCs indeed express SHANK3, we used a specific GCs marker, Brn3a, to perform double-labeling with SHANK3 (Sato et al., 2017; Christiansen et al., 2018). The merged image (Figure 6a”) reveals that the Brn3a-positive GCs in the GCL expressed SHANK3 because the two label colors (somata, stained red by Brn3a; membranes, stained green by SHANK3) perfectly fit into each other. Our results demonstrated that 411 GCs with a strong immunofluorescence signal for Brn3a were observed in 30 retinal slices, and all of them were SHANK3-positive (100% positive rate). However, a few SHANK3-positive cells were not double-labeled with Brn3a (arrowheads in Figures 6a–a”). These cells could be Brn3a-negative GCs or ectopic ACs.

### SH3 and Multiple Ankyrin Repeat Domains Protein 3 Is Expressed in the Müller Cells

The double labeling of SHANK3 with CRALBP, a specific marker for labeling both the somata and processes of mice Müller cells, was performed to assess the expression of SHANK3 in the Müller cells (Kolesnikov et al., 2021). These cells spanned the entire retina, with their somata distributed in the INL, and their processes expanded to the GCL to form characteristic end-feet (Figure 6b’). As shown in Figures 6b–b”, SHANK3 was strongly expressed in the soma of the Müller cells (arrows). A total of 795 CRALBP-labeled neurons in 30 retinal slices were identified in Müller cells (795 out of 795 cells from 30 retinal slices, with a 100% positive rate), but the main trunks, distinguishable parallel processes, and end-feet in the GCL could not be labeled with SHANK3.

## Discussion

The gene mutation of SHANK3 leads to PMS and causes visual problems (Phelan et al., 2005). However, we still barely understand the distribution and function of SHANK3 in the retina. In this study, using double-labeling experiments, we found that SHANK3 is broadly expressed throughout the inner and outer mice retina. In the outer retina, SHANK3 was expressed in the somata of blue cones (marked by S-opsin, Figures 2a,a”), OS of red/green cones (marked by L/M-opsin, Figures 2b,b”) and OS of rods (marked by Rho4D2, Figures 2c,c”), HCs (marked by CB, Figures 3a,a”), and BCs (marked by ChX10, Figures 3b,b”) as well as in the OPL. These results suggest that SHANK3 not only exists in the somata of HCs and BCs but also may be located in the processes of these cells. This highlights the possibility of SHANK3 contributing to lateral retinal signaling regulation. In the inner retina, SHANK3-positive IR was found in the ACs (marked by GAD65, TH, and ChAT, Figure 4) and GCs (marked by Bin3a, Figures 6a,a”), and it was also expressed in the IPL, which means SHANK3’s expression may take part in longitudinal retinal information regulation. Meanwhile, the somata of the Müller cells (marked by CRALBP, Figures 6b,b”), unlike their processes and end-feet, were also co-labeled with SHANK3. It is well known that Müller cells are the principal glial cells of the retina, and they are responsible for the maintenance of the homeostasis of the retinal extracellular milieu (Bringmann et al., 2009). That means SHANK3 may also take a part in the neuromodulate effects of glia. Despite our efforts to optimize experimental conditions, the immunostaining for SHANK3 did not exhibit cell-type specificity. We found that in Huang’s work, the SHANK3 antibody (from Santa Cruz, sc377470) shows a characteristic staining pattern in the mouse cortex (Huang et al., 2019); therefore, we tested the specificity of this new antibody expression in the mouse retina by western blot and immunohistochemistry but the results showed that the specificity of this new antibody in the retina was poor (data not shown). Perhaps new antibodies will become available in the future, allowing to reveal a cell-type-specific pattern of staining for SHANK3 in the retina.

Autism spectrum disorder constitutes a group of neurodevelopmental disorders, some of which are caused by synaptic impairment (Bourgeron, 2015). SHANK3 has been found to be located at the excitatory synapses of the rat hippocampus (Naisbitt et al., 1999; Sheng and Hoogenraad, 2007) and is part of the glutamate receptosome that physically link ionotropic N-methyl-D-aspartic acid (NMDA) receptors to metabotropic glutamate receptor 5 (mGlu5) through interactions with scaffolding proteins PSD-95-guanylate kinase-associated protein (GKAP)-SHANK3-Homer (Moutin et al., 2021). The model of *Shank3*-mutant mice has exhibited molecular impairments in the cortex and hippocampus neurons, causing deficits in the NMDA receptor-mediated neurotransmission associated with cognitive deficits (Kouser et al., 2013; Duffney et al., 2015; Sala et al., 2015; Qin et al., 2018). Because of the important relationship between mGlu5 and NMDA receptors in the induction of synaptic plasticity, when *Shank3* is mutant, the destruction of the scaffolding complex makes the mGlu5 and NMDA receptors of the hippocampal neurons interact directly, with the reciprocal function inhibited and the activity of the synaptic NMDA receptor also decreased. This then limits the efficacy of the synaptic transmission and prevents the induction of synaptic plasticity (Perroy et al., 2008; Moutin et al., 2012, 2021). Some previous works have found that, in a *Shank2*^–/–^ ASD model, the NMDA/a-amino-3-hydroxyl-5-methyl-4-isoxazole-propionic acid (AMPA) current ratio in the hippocampus is increased when compared to that in a WT model (Schmeisser et al., 2012; Won et al., 2012); meanwhile, in the *Shank3*^–/–^ ASD model, the NMDA/AMPA current ratio in the hippocampus is decreased compared to in a WT model (Moutin et al., 2021). This also affects the AMPA signaling transmission in the cortex and striatal when shank genes are deleted (Monteiro and Feng, 2017). Therefore, whether SHANK3 plays a role in the synaptic plasticity of the mouse retina requires further study (Bozdagi et al., 2010).

## Conclusion

Our results identified a large number of prominent cells that expressed high immunoreactive levels of SHANK3 in the retina, particularly in the GCL and INL. SHANK3 positive signals located in cones, rods, HCs, and BCs, suggested that SHANK3 might participate in the lateral signaling pathway among photoreceptors, HCs, and BCs. SHANK3 were also expressed in several subtypes of ACs indicated that they might mediate the longitude signaling transmission in INL. As we know, GCs are the necessary neurons for retinal signal output. The expression of SHANK3 in GCs suggested that SHANK3 might be involved in retinal signal output. SHANK3 were also located in Müller cells and might participate in the glia neuromodulate functions. The wide expression patterns of SHANK3 in mice retina indicate that SHANK3 may play an important role in the visual signaling pathway.

## Data Availability Statement

The original contributions presented in the study are included in the article/Supplementary Material, further inquiries can be directed to the corresponding author/s.

## Ethics Statement

The animal study was reviewed and approved by the National Institutes of Health (NIH) guidelines for the Care and Use of Laboratory Animals and the regulations of Anhui Medical University regarding the ethical use of animals.

## Author Contributions

PZ and LW designed the research, analyzed the data, and wrote the manuscript. YX, YW, and GT mainly performed the research. LL, JC, LZ, and QX partly performed the research and analyzed the data. All authors made valuable comments and edits to the manuscript, read and approved the final version of the manuscript.

## Conflict of Interest

The authors declare that the research was conducted in the absence of any commercial or financial relationships that could be construed as a potential conflict of interest.

## Publisher’s Note

All claims expressed in this article are solely those of the authors and do not necessarily represent those of their affiliated organizations, or those of the publisher, the editors and the reviewers. Any product that may be evaluated in this article, or claim that may be made by its manufacturer, is not guaranteed or endorsed by the publisher.
