# Supplementary material for: Expression of SH3 and Multiple Ankyrin Repeat Domains Protein 3 in Mouse Retina
Source: Front Cell Neurosci. 2022 Mar 25;16:795668. doi: 10.3389/fncel.2022.795668 (PMC8990853; doi:10.3389/fncel.2022.795668)
Supplement: Supplementary file 1 [file Data_Sheet_1.PDF]

Expression of SHANK3 in mouse retina

Yue. Xu[1]*, Ya'nan. Wang[1]*, Guang'an. Tong[2]*, Lin. Li[1], Juan. Cheng[1], Lesha. Zhang[1], Qi. Xu[1], Liecheng. Wang[1]# and Pingping. Zhang[1]#

[1] Department of Physiology, School of Basic Medical Sciences, Anhui Medical University, Hefei 230032, China

[2] Department of Neurology, The Affiliated Hospital of the Neurology Institute, Anhui University of Chinese Medicine, Hefei 230061, China

***These authors contributed equally:** Yue. Xu, Ya'nan. Wang, Guang'an. Tong

# Correspondence:

Dr. Pingping. Zhang, Department of Physiology,School of Basic Medical Sciences,Anhui Medical University, 81 Meishan Road, Hefei, Anhui, China, 791663310@qq.com

Dr. Liecheng. Wang, Department of Physiology,School of Basic Medical Sciences,Anhui Medical University, 81 Meishan Road, Hefei, Anhui, China, wangliecheng@ahmu.edu.cn

Keywords: ASD$_1$, SHANK3$_2$, Retina$_3$, Double-labeled immunohistochemistry$_4$, Excitatory synapse$_5$.

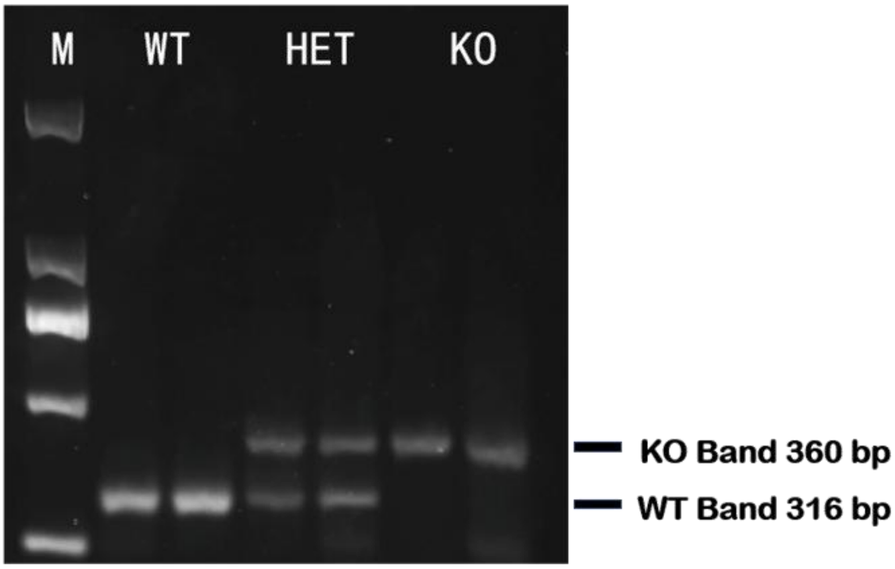

Supplementary Figure 1. Genotype analyses of shank3b KO mice. Ethidium bromide-stained agarose gel with PCR reactions from three different mice tails (WT, HET and KO). KO represents *shank3b* KO mice with a DNA double-strand length of 360 bp. HET represents heterozygote mice with two DNA double-strands, which length are 316 bp and 360 bp. WT represents WT mice with a DNA double-strand length of 131bp. M: marker.

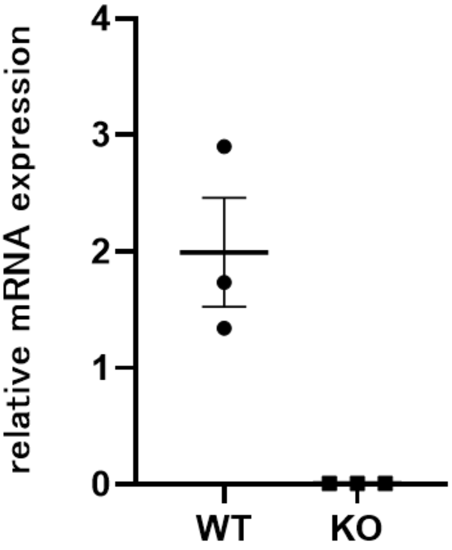

Supplementary Figure 2. Quantitative PCR analysis of SHANK3 mRNA in WT and shank3b knockout mice. GAPDH (Gene ID: 14433), Forward Sequence: CATCACTGCCACCCAGAAGACTG; Reverse Sequence: ATGCCAGTGAGCTTCCCGTTCAG. shank3 (Gene ID: 58234), Forward Sequence: ACCTTGAGTCTGTAGATGTGGAAG; Reverse Sequence: GCTTGTGTCCAACCTTCACGAC.

	WT			KO		
SHANK3	28.02	28.34	27.33	33.76	34.39	33.66
	28.05	28.11	27.95	32.88	33.97	34.15
	28.20	27.56	27.4	33.51	32.97	33.1
	29.20	27.48	27.76	33.27	32.45	34.45
GAPDH	16.09	16.05	16.69	13.45	13.96	14.33
	15.91	15.9	16.26	13.6	13.51	14.05
	16.25	15.89	16.07	13.7	13.57	13.99
	15.89	15.8	16.55	14.18	13.44	13.56

Supplementary Table 1. Quantitative PCR analysis of SHANK3 mRNA in WT and shank3b knockout mice. Each column in the table represents a sample.
